# Serum pentraxin 3 as a biomarker of hepatocellular carcinoma in chronic hepatitis B virus infection

**DOI:** 10.1038/s41598-020-77332-3

**Published:** 2020-11-20

**Authors:** Huan Deng, Xiude Fan, Xiaoyun Wang, Lu Zeng, Kun Zhang, Xiaoge Zhang, Na Li, Qunying Han, Yi Lv, Zhengwen Liu

**Affiliations:** 1 Department of Infectious Diseases, First Affiliated Hospital of Xi’an Jiaotong University, No. 277 Yanta West Road, Xi’an, 710061 Shaanxi Province People’s Republic of China; 2 Department of Hepatobiliary Surgery, First Affiliated Hospital of Xi’an Jiaotong University, Xi’an, 710061 Shaanxi People’s Republic of China; 3 Institute of Advanced Surgical Technology and Engineering, Xi’an Jiaotong University, Xi’an, 710061 Shaanxi People’s Republic of China

**Keywords:** Cancer, Immunology, Microbiology, Biomarkers, Diseases, Oncology

## Abstract

Biomarkers for early diagnosis of hepatocellular carcinoma (HCC) are needed in chronic hepatitis B virus (HBV) infection, a leading cause of HCC. We evaluated whether measurement of serum pentraxin 3 (PTX3) could improve diagnosis of HCC in chronic HBV infection. Data from patients with HBV-related chronic hepatitis (n = 159), cirrhosis (n = 99) and HCC (n = 107), and healthy controls (n = 151) were analyzed. Serum PTX3 concentration was measured by immunoassay. Area under the receiver operating characteristic curve (AUC) was applied to assess diagnostic accuracy. PTX3 levels were significantly higher in HBV patients than in healthy controls (*P* < 0.001) and in HCC than in chronic hepatitis (*P* < 0.001) or cirrhosis patients (*P* < 0.001). PTX3 was an independent risk factor of HCC [odds ratio (OR) 1.617, *P* < 0.001] and could distinguish HCC in chronic HBV infection [cutoff 9.231 ng/mL, AUC 0.929 with 95% confidence interval (CI) of 0.898–0.953], including α-fetoprotein (AFP) negative [cutoff 8.985 ng/mL, AUC (95%CI) 0.947 (0.908–0.973)] and early-stage HCC [cutoff 9.359 ng/mL, AUC (95%CI) 0.920 (0.885–0.947)]. Combination of PTX3 with AFP improved the discrimination of early HCC from chronic HBV infection [AUC (95%CI) 0.948 (0.918–0.970)]. In short, PTX3 measurement could identify HCC, including AFP-negative and early-stage HCC, in chronic HBV infection.

## Introduction

Hepatocellular carcinoma (HCC) is the sixth most common cancer and the second leading cause of cancer-related deaths worldwide with more than 840,000 new cases each year^1^. HCC is also the major cause of death among patients with cirrhosis^2^. Chronic hepatitis B virus (HBV) infection, which is associated with various liver diseases including chronic hepatitis, cirrhosis and HCC^3–5^, is the principal risk factor for HCC in East Asian counties including China^6,7^. The identification of HCC, especially at the early stage, from the various disease conditions in chronic HBV infection is of vital importance for improving patient prognosis because that early diagnosis is considered to be the only chance for long-term survival of HCC patients^7–9^.

The commonly used modalities for detection of HCC include determination of serum alpha-fetoprotein (AFP), abdominal ultrasound, computed tomography and magnetic resonance imaging^10^. However, the differentiation of small HCC from cirrhotic nodules is still difficult by imaging. Meanwhile, AFP, the most commonly used biomarker in HCC, is not specific for HCC and elevated levels of AFP may be seen in both patients with HCC and chronic hepatitis without cancer^11–13^. In addition, AFP is potentially most suitable to detect advanced tumors because the concentrations are related to tumour size^11^. Moreover, many HCC patients may exhibit no elevation of AFP levels and AFP determination can not be utilized to detect HCC in these cases^12^. Therefore, identification of new biomarkers for diagnosis, especially early diagnosis, of HCC is an urgent need for clinical practice^14^.

Pentraxin 3 (PTX3), also called tumor necrosis factor-stimulated gene 14 (TSG-14), is a long pentraxin of the pentraxin superfamily^15,16^, a class of ancient group of evolutionarily conserved and versatile proteins^17,18^. Structurally, PTX3 has an unrelated N-terminal domain linked to a pentraxin-like C-terminal domain^19,20^. PTX3 performs indispensable non-redundant roles in humoral innate immunity in microbial infections and acts as a connection between innate immunity, inflammation, tissue repair and cancer^17,21–24^. Notably, PTX3 is possibly involved in cancer development. For example, PTX3 expression levels have been shown to be related to the prognosis in certain types of cancer such as breast cancer^25^, gastric cancer^26^, lung carcinoma^27^, pancreatic carcinoma^28^, and prostate cancer^29^.

With regard to liver diseases, plasma PTX3 was indicated to be associated with nonalcoholic steatohepatitis (NASH)^30^ and could differentiate NASH from non-NASH^31^. Elevated levels of PTX3 were also documented in acute liver injury caused by paracetamol and linked with adverse consequences in paracetamol overdose^32^. Hepatic and plasma PTX3 was raised in alcoholic hepatitis patients and PTX3 expression was related to the disease severity and short-term mortality^33^. Serum PTX3 was clinically shown to have valid diagnostic accuracy as a marker of fibrosis in chronic hepatitis C virus (HCV) infection^34^ and increased PTX3 plasma level was a risk factor for HCC occurrence in chronic HCV infection^35^. Furthermore, PTX3 was shown to be able to promote HCC progression and high PTX3 expression in tumor tissues was related to unfavorable prognosis in HCC patients^36^. Despite these studies, the potential role of circulating PTX3 levels in chronic HBV infection, especially HBV-related HCC, remains to be further examined. This study, therefore, was designed to determine the serum PTX3 levels in patients with various cross-sectional diseases including chronic hepatitis, cirrhosis and HCC during chronic HBV infection and to assess the potential diagnostic value in distinguishing HCC, including AFP-negative and early HCC, from other disease conditions in chronic HBV infection.

## Methods

### Study subjects

A total of 516 participants were included in this study. Among them, 365 were patients with chronic HBV infection (159 chronic hepatitis, 99 cirrhosis and 107 HBV-related HCC) and 151 were age- and sex-matched healthy controls. Chronic HBV infection and liver diseases were diagnosed in accordance with Clinical Practice Guidelines on the management of HBV infection^37^. Measurement of AFP level and ultrasound sonography were routinely performed in all the patients with chronic HBV infection. Chronic HBV infection was defined based on the presence of HBsAg for more than 6 months and detectable serum HBV DNA levels. Chronic hepatitis was diagnosed in view of abnormal biochemistry of liver function such as increased serum alanine aminotransferase (ALT) and aspartate aminotransferase (AST) levels in chronic HBV infection. Cirrhosis was determined based on imaging evidence of liver cirrhosis and existence of portal hypertension, ascites, varices and splenomegaly and/or changes of histopathology of liver biopsy samples with abnormal biochemistry of liver function such as increased ALT and AST levels and/or hypoalbuminia. HCC was determined on the base of characteristics of ultrasound, computed tomography and/or magnetic resonance and/or histopathology^38^. According to the commonly used upper limit of normal AFP level (20 ng/mL)^38^, the HCC was divided into AFP negative (≤ 20 ng/mL) or positive (AFP > 20 ng/mL) HCC. HCC stage was defined in line with the Barcelona Clinic Liver Cancer (BCLC) staging system. The diseases were classified as early-stage (stage 0 and stage A)^39^ and late-stage (stage B, stage C and stage D) HCC. Patients with other liver disease such as nonalcoholic fatty liver disease, primary biliary cirrhosis, alcoholic liver disease and drug-induced liver injury were excluded. Patients with autoimmune diseases such as autoimmune hepatitis and systemic lupus erythematosus and severe diseases of other systems were also excluded. Patients aged under 18 years were also excluded. Blood samples were obtained from each participant. Serum was separated, aliquoted and stored at—80 °C until use.

### Determination of laboratory parameters

Blood tests and liver functions were assayed at the central laboratory of the hospital. Serum HBV DNA was quantified by HBV quantitative polymerase chain reaction. HBsAg, anti-HBs, HBeAg, anti-HBe, and anti-HBc were determined by enzyme-linked immunosorbent assay. Serum AFP (ng/mL) was quantified by automated Eleceyes (Hoffman-La Roche Ltd., Basel, Switzerland).

### Measurement of serum PTX3 concentration

Serum PTX3 concentration was estimated by The Quantikine Human Pentraxin 3/TSG-14 Immunoassay (R&D Systems China Co., Ltd. Shanghai, China). The intra-assay precision [coefficient of variation CV (%)] is 3.6% to 4.4%. The inter-assay precision is 4.1% to 6.2%. The minimum detectable dose (MDD) of PTX3 is 0.007–0.116 ng/mL. The mean MDD is 0.025 ng/mL. No significant crossreactivity or interference was observed with human C-reactive protein and Pentraxin 2/Serum amyloid P component.

### Statistical analysis

Statistical analysis was conducted using SPSS 24.0 software and MedCalc12.0 software. Quantitative variables were presented as mean and standard deviation (SD) or median and interquartile range. Categorical variables were expressed as absolute or relative frequencies. Continuous variables were compared using the analysis of variance. Categorical variables were compared by Chi-Square test. Risk factors for HCC were estimated using multivariable logistic regression analysis. The predictive values of relevant parameters were analyzed by receiver operating characteristic (ROC) curve to calculate the area under the curve (AUC), 95% confidence interval (CI) and optimal cut-off value and its sensitivity, specificity, Yoden index, positive (PPV) and negative (NPV) predictive value, and positive and negative likelihood ratio (LR). Analysis of statistical differences between AUCs was performed using Z-test. To minimize the influence of data bias and confounding variables we also performed analysis of the data for patients with similar propensity scores using a 1:1 ratio matching after propensity score matching (PSM). Significance was defined as a two-tailed *P* value less than 0.05.

### Ethical approval

This study was conducted in conform with the Declaration of Helsinki. Study approval was obtained from the Ethics Committee of the First Affiliated Hospital of Xi'an Jiaotong University. Each study participant gave informed consent.

## Results

### Characteristics of the participants

This study recruited 516 participants including 365 patients with chronic HBV infection [male/female, 249/116; age, 41.31 ± 13.69 (18–78) years] and 151 healthy controls [male/femal, 97/54; age, 42.28 ± 14.13 (18–76) years]. The male/female ratio and age between patients with chronic HBV infection and healthy controls were comparable (*P* > 0.05). Of the 365 patients, 159 (43.6%) were diagnosed with chronic hepatitis, 99 (27.1%) with cirrhosis and 107 (29.3%) with HCC. The demographic and laboratory findings in the patients with different diseases were shown in Table 1.

**Table 1:** Demographic and laboratory parameters in the patients with different clinical diseases of chronic HBV infection.

	Chronic hepatitis (n = 159)	Cirrhosis (n = 99)	HCC (n = 107)	*P*
Gender (male/female)	96/63	61/38	92/15	< 0.001
Age (years)	32.30 ± 12.39	46.98 ± 9.18	49.44 ± 10.90	< 0.001
HBV DNA (IU/mL, log)	5.92 ± 1.83	5.34 ± 1.62	4.90 ± 1.50	< 0.001
ALT (IU/L)	81 (31–331)	48 (28–84)	54 (33–102)	0.003
AST (IU/L)	65 (29–152)	58 (37–83)	70 (35–150)	0.538
TBIL (μmol/L)	18.5 (11.4–84.8)	26.1 (16.2–44.7)	26.2 (15.2–46.7)	0.108
Albumin (g/L)	38 (34.9–41)	30 (26.8–36.2)	33 (29.7–39.1)	< 0.001
AFP (ng/mL)	12 (6.8–26.2)	12 (4.82–42)	97.4 (16.8–4886)	< 0.001
PTX3 (ng/mL)	3.7 (1.6–6.4)	6.2 (3–9)	15.6 (9.6–24.3)	< 0.001

*HBV* hepatitis B virus, *HCC* hepatocellular carcinoma, *ALT* alanine aminotransferase, *AST* aspartate aminotransferase, *TBIL* total bilirubin, *PTX3* pentraxin 3, *AFP* α-fetoprotein.

### Serum PTX3 levels in the study participants

Serum PTX3 levels in patients with chronic HBV infection [6.63 (3.02–10.24) ng/mL] were significantly increased compared with healthy controls [1.03 (0.6–1.7) ng/mL, *P* < 0.001] (Figure S1A). HCC patients had significantly increased PTX3 levels [15.6 (9.6–24.3) ng/mL] compared with patients with chronic hepatitis [3.7 (1.6–6.4) ng/mL, *P* < 0.001, Figure S1B], cirrhosis [6.2 (3–9) ng/mL, *P* < 0.001, Figure S1B,] or chronic HBV infection without HCC [chronic hepatitis + cirrhosis, 4.2 (2–7.9) ng/mL, *P* < 0.001, Figure S1C].

According to underlying liver disease, patients with chronic hepatitis and HCC (n = 23) had significantly higher PTX3 levels than patients with chronic hepatitis without HCC (n = 159) [13.3 (6.8–38.8) ng/mL vs. 3.7 (0.089–22.37) ng/mL, *P* < 0.001, Figure S2A]. Patients with cirrhosis and HCC (n = 84) also had significantly higher PTX3 levels than patients with cirrhosis without HCC (n = 99) [15.9 (1.3–56.6) ng/mL vs. 6.18 (0.83–14.02) ng/mL, *P* < 0.001, Figure S2B].

### Association of PTX3 levels with HCC in chronic HBV infection

The patients were categorized as chronic hepatitis, cirrhosis and chronic HBV infection without HCC (chronic hepatitis plus cirrhosis). Multivariate logistic regression on factors for HCC showed that, in comparison with chronic hepatitis, in addition to gender (OR 19.288, *P* = 0.003), age (OR 1.137, *P* < 0.001) and AFP (OR 1.001, *P* = 0.014), PTX3 (OR 1.639, *P* < 0.001) was an independent risk factor for HCC (Table 2). In relation to cirrhosis, together with gender (OR 5.612, *P* = 0.041), age (OR 1.061, *P* = 0.047), HBV DNA (OR 0.619, *P* = 0.022) and albumin (OR 0.909, *P* = 0.026), PTX3 (OR 1.671, *P* < 0.001) was an independent risk factor for HCC (Table 2). In relation to HBV infection without HCC, in addition to gender (OR 14.364, *P* = 0.003), age (OR 1.100, *P* < 0.001), HBV DNA (OR 0.672, *P* = 0.015) and AFP (OR 1.001, *P* = 0.002), PTX3 (OR 1.617, *P* < 0.001) was also an independent risk factor for HCC (Table 2).

**Table 2:** Factors associated with HCC by multivariate logistic regression.

Variable	HCC versus CH (n = 107) versus (n = 159)			HCC versus LC (n = 107) versus (n = 99)			HCC versus CH + LC (n = 107) versus (n = 258)		
	OR	SE	*p*	OR	SE	*p*	OR	SE	P
Gender (Male/female)	19.288	0.995	0.003	5.612	0.844	0.041	14.364	0.906	0.003
Age (years)	1.137	0.032	< 0.0001	1.061	0.030	0.047	1.100	0.024	< 0.0001
HBV DNA (IU/mL, log)	0.656	0.234	0.072	0.619	0.210	0.022	0.672	0.163	0.015
ALT (IU/L)	0.996	0.004	0.243	0.999	0.005	0.767	0.997	0.003	0.303
AST (IU/L)	1.003	0.004	0.421	1.002	0.003	0.570	1.002	0.002	0.388
TBIL (μmol/L)	0.997	0.003	0.330	1.002	0.005	0.687	1.000	0.003	0.979
Albumin (g/L)	0.995	0.061	0.930	0.909	0.043	0.026	0.942	0.038	0.120
PTX3 (ng/mL)	1.639	0.097	< 0.0001	1.671	0.104	< 0.0001	1.617	0.075	< 0.0001
AFP (ng/mL)	1.001	0.0002	0.014	1.003	0.002	0.193	1.001	0.0002	0.002

*HBV* hepatitis B virus, *HCC* hepatocellular carcinoma, *ALT* alanine aminotransferase, *AST* aspartate aminotransferase, *TBIL* total bilirubin, *PTX3* pentraxin 3, *AFP* α-fetoprotein, *CH* chronic hepatitis, *LC* liver cirrhosis.

Of the HCC patients, 43 patients had histological results of HCC. Of the 43 patients, 17 had low differentiation, 19 had moderate differentiation and 7 had high differentiation. The PTX3 levels in patients with low differentiation were significantly higher than in patients with high differentiation [20.57(8.47–56.62) ng/mL vs. 7.93(5.95–9.03) ng/mL, *P* < 0.001]. Patients with moderate differentiation also had higher PTX3 levels than patients with high differentiation [15.10 (7.78–42.18) ng/mL vs. 7.93(5.95–9.03) ng/mL, *P* = 0.001]. The PTX3 levels in patients with low differentiation were higher than in patients with moderate differentiation [20.57(8.47–56.62) ng/mL vs. 15.10 (7.78–42.18) ng/mL] although the difference was not significant (*P* = 0.383).

### Diagnostic performance of serum PTX3 levels for HCC

**Figure 1:**
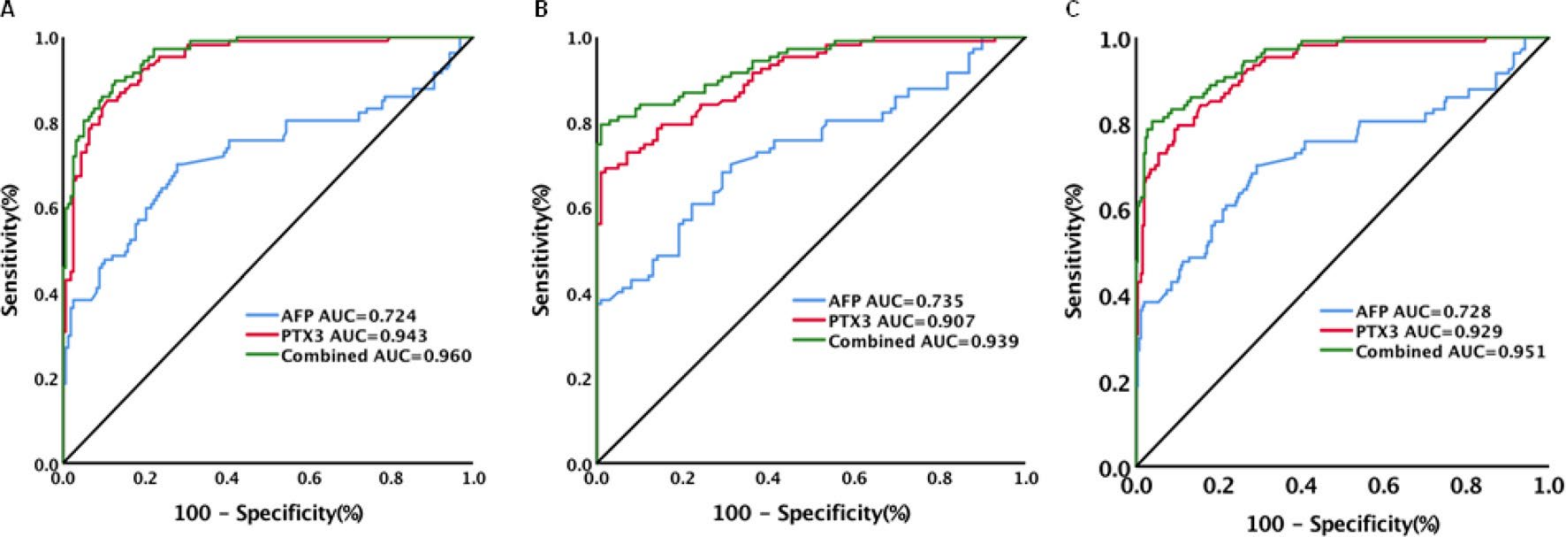
Diagnostic performance of serum PTX3, AFP and combination of PTX3 with AFP for hepatocellular carcinoma (HCC) in patients with chronic HBV infection. (**A**) Diagnostic performance of serum PTX3, AFP and combination of PTX3 with AFP for HCC from chronic hepatitis; (**B**) Diagnostic performance of serum PTX3, AFP and combination of PTX3 with AFP for HCC from cirrhosis; (**C**) Diagnostic performance of serum PTX3, AFP and combination of PTX3 with AFP for HCC from patients without HCC (chronic hepatitis and cirrhosis).

**Figure 2:**
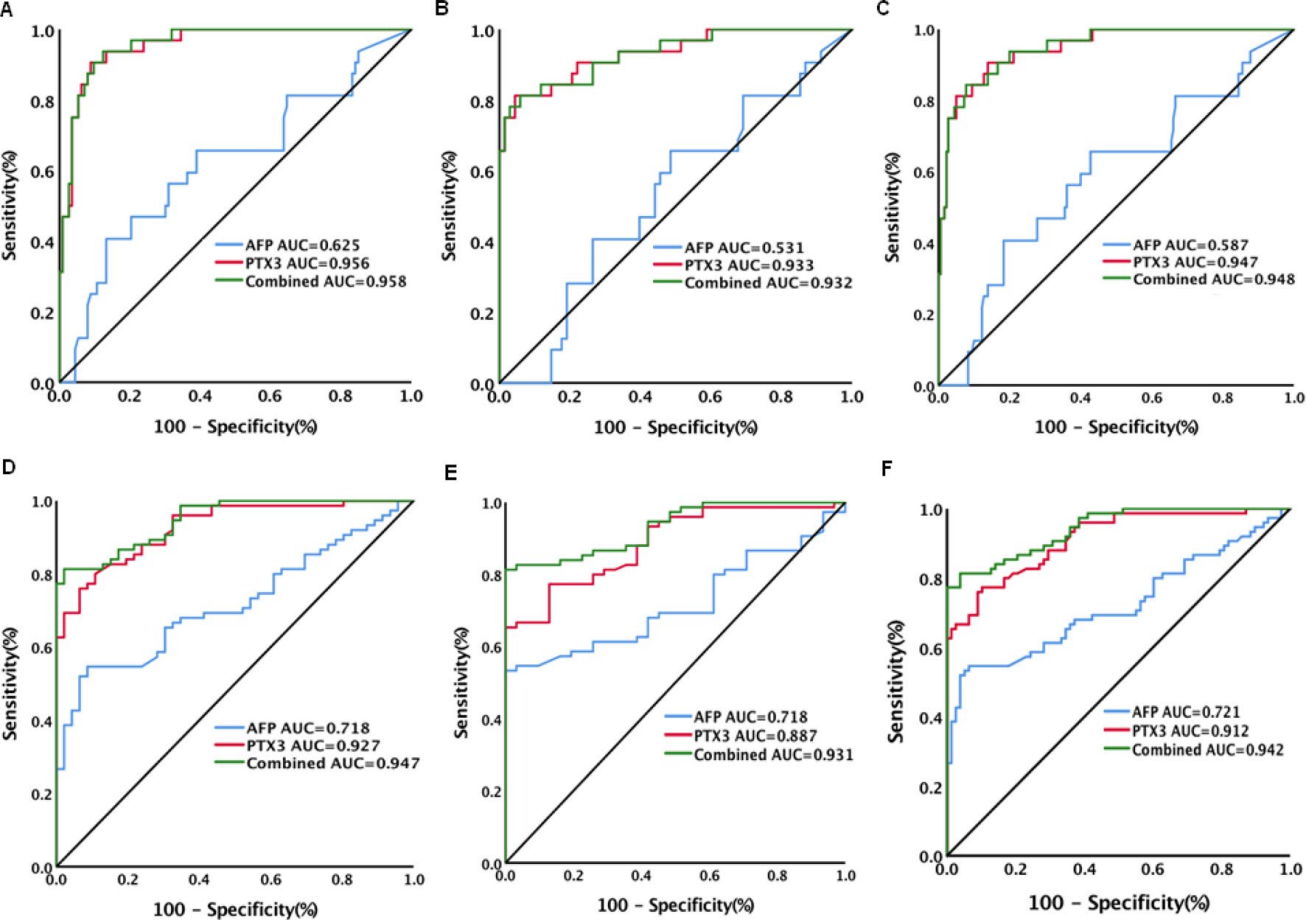
Diagnostic performance of serum PTX3, AFP and combination of PTX3 with AFP for AFP negative (≤ 20 ng/mL) and positive (> 20 ng/mL) hepatocellular carcinoma (HCC) in patients with chronic HBV infection. (**A**) Diagnostic performance of serum PTX3, AFP and combination of PTX3 with AFP for AFP negative HCC from chronic hepatitis; (**B**) Diagnostic performance of serum PTX3, AFP and combination of PTX3 with AFP for AFP negative HCC from cirrhosis; (**C**) Diagnostic performance of serum PTX3, AFP and combination of PTX3 with AFP for AFP negative HCC from patients without HCC (chronic hepatitis and cirrhosis); (**D**) Diagnostic performance of serum PTX3, AFP and combination of PTX3 with AFP for AFP positive HCC from chronic hepatitis; (**E**) Diagnostic performance of serum PTX3, AFP and combination of PTX3 with AFP for AFP positive HCC from cirrhosis; (**F**) Diagnostic performance of serum PTX3, AFP and combination of PTX3 with AFP for AFP positive HCC from patients without HCC (chronic hepatitis and cirrhosis).

The performance of PTX3 for identifying HCC was assessed. Serum PTX3 levels could significantly differentiate HCC from chronic hepatitis [AUC (95% CI) 0.943 (0.908–0.968), sensitivity 85.1%, specificity 89.3%, Fig. 1A], cirrhosis [AUC (95% CI) 0.907 (0.859–0.943), sensitivity 68.2%, specificity 99.0%, Fig. 2B] or chronic HBV infection without HCC [AUC (95% CI) 0.929 (0.898–0.953), sensitivity 79.4%, specificity 89.9%, Fig. 2C, Table 3). The corresponding results of AFP were: HCC versus chronic hepatitis: AUC (95% CI) 0.724 (0.666–0.776), sensitivity 70.1%, specificity 72.3% (Fig. 2A); HCC versus cirrhosis: AUC (95% CI) 0.735 (0.670–0.794), sensitivity 68.2%, specificity 70.7% (Fig. 1B); and HCC versus chronic HBV infection without HCC: AUC (95%CI) 0.728 (0.679–0.773), sensitivity 70.1%, specificity 70.5% (Fig. 1C). The combination of PTX3 with AFP improved the performance of PTX3 or AFP alone. The corresponding results of combination of PTX3 with AFP were: HCC versus chronic hepatitis: AUC (95%CI) 0.960 (0.929–0.980), sensitivity 89.7%, specificity 87.4% (Fig. 1A); HCC versus cirrhosis: AUC (95%CI) 0.939 (0.898–0.968), sensitivity 79.4%, specificity 99.0% (Fig. 1B); and HCC versus chronic HBV infection without HCC: AUC (95%CI) 0.951 (0.924–0.971), sensitivity 80.4%, specificity 96.1% (Fig. 1C, Table 3).

**Table 3:** Diagnostic performance of serum PTX3, AFP and the combination of PTX3 with AFP levels for HCC in chronic hepatitis, cirrhosis and chronic HBV infection without HCC (CH + LC).

	AUC (95% CI)	Cutoff (ng/mL)	Sensitivity (%)	Specificity (%)	Youden index	PPV (%)	NPV (%)	Positive LR	Negative LR
HCC versus CH									
PTX3	0.943 (0.908–0.968)	8.949	85.1	89.3	0.744	84.3	89.9	7.95	0.17
AFP	0.724 (0.666–0.776)	20.36	70.1	72.3	0.424	63	78.2	2.53	0.41
PTX3 + AFP	0.960 (0.929–0.980)	-	89.7	87.4	0.771	82.8	92.7	7.13	0.12
HCC versus LC									
PTX3	0.907 (0.859–0.943)	11.719	68.2	99.0	0.672	98.6	74.2	67.54	0.32
AFP	0.735 (0.670–0.794)	22.22	68.2	70.7	0.389	71.6	67.3	2.33	0.45
PTX3 + AFP	0.939 (0.898–0.968)	-	79.4	99.0	0.784	98.8	81.7	78.6	0.21
HCC versus CH + LC									
PTX3	0.929 (0.898–0.953)	9.231	79.4	89.9	0.693	76.6	91.3	7.88	0.23
AFP	0.728 (0.679–0.773)	20.36	70.1	70.5	0.406	49.7	85	2.38	0.42
PTX3 + AFP	0.951 (0.924–0.971)	-	80.4	96.1	0.765	89.6	92.2	20.74	0.2

*PTX3* pentraxin 3, *AFP* alpha-fetoprotein, *HBV* hepatitis B virus, *HCC* hepatocellular carcinoma, *CH* chronic hepatitis, *LC* liver cirrhosis, *AUC* area under receiver operating characteristic (ROC) curve, *PPV* positive predictive value, *NPV* negative predictive value, *LR* likelihood ratio.

After PSM, all the parameters (except PTX3 levels) in HCC (n = 44) versus chronic hepatitis (n = 44), HCC (n = 61) versus cirrhosis (n = 61) and HCC (n = 80) versus non-HCC (n = 80) were well matched (Table S1). Analysis of the data after PSM yielded similar results. The AUCs in HCC versus chronic hepatitis, HCC versus cirrhosis and HCC versus non-HCC were 0.954 (0.887–0.987), 0.914 (0.849–0.957) and 0.936 (0.887–0.969), respectively (Table S2).

Among the patients with chronic HBV infection, 87 patients received antiviral treatment with nucleos(t)ide analogues for more than six months and 278 did not receive antiviral treatment (Table S3). PTX3 levels could highly discriminate HCC in patients under or not under antiviral treatment. In patients under antiviral treatment, PTX3 levels had an AUC (95%CI) of 0.923 (0.865–0.982), sensitivity of 80%, and specificity of 89.47% for HCC in chronic HBV infection without HCC. AFP levels had an AUC (95%CI) of 0.731 (0.609–0.852), sensitivity of 43.33%, and specificity of 100%. The combination of PTX3 and AFP had an AUC (95%CI) of 0.947 (0.900–0.994), sensitivity of 76.67% and specificity of 98.25% (Figure S3A, Table S4). In patients not under antiviral treatment, PTX3 levels had an AUC (95%CI) of 0.929 (0.896–0.963), sensitivity of 79.22%, and specificity of 91.54%. AFP levels had an AUC (95%CI) of 0.729 (0.653–0.804), sensitivity of 68.83% and specificity of 74.13%. The combination of PTX3 and AFP had an AUC (95%CI) of 0.952 (0.927–0.977), sensitivity of 80.52% and specificity of 96.02% (Figure S3B, Table S4).

According to AFP levels (Table S5), PTX3 levels remained highly discriminative of AFP negative HCC from chronic hepatitis [AUC (95%CI) 0.956 (0.909–0.983), sensitivity 90.6%, specificity 91.2%, Fig. 2A], cirrhosis [AUC (95%CI) 0.933 (0.865–0.973), sensitivity 81.3%, specificity 95.6%, Fig. 2B] and chronic HBV infection without HCC [AUC (95%CI) 0.947 (0.908–0.973), sensitivity 90.6%, specificity 86.1%, Fig. 2C, Table S6]. The corresponding results of AFP were: HCC versus chronic hepatitis: AUC (95%CI) 0.625 (0.540–0.704), sensitivity 40.6%, specificity 86.7% (Fig. 2A); HCC versus cirrhosis: AUC (95%CI) 0.531 (0.429–0.632), sensitivity 65.6%, specificity 51.5% (Fig. 2B); and HCC versus chronic HBV infection without HCC: AUC (95%CI) 0.587 (0.518–0.654), sensitivity 65.6%, specificity 57.2% (Fig. 2C, Table S4). The addition of AFP to PTX3 did not significantly improve the performance of PTX3 for discriminating AFP negative HCC. Analysis in patients with AFP positive (> 20 ng/mL) HCC showed that PTX3 levels were also highly discriminative of HCC from chronic hepatitis [AUC (95%CI) 0.927 (0.865–0.966), sensitivity 76%, specificity 93.5%, Fig. 2D], cirrhosis [AUC (95%CI) 0.887 (0.810–0.940), sensitivity 65.3%, specificity 100%, Fig. 2E] and chronic HBV infection without HCC [AUC (95%CI) 0.912 (0.855–0.952), sensitivity 77.3%, specificity 89.7%, Fig. 2E, Table S6]. The corresponding results of AFP were: HCC versus chronic hepatitis: AUC (95%CI) 0.718 (0.629–0.796), sensitivity 54.7%, specificity 91.3% (Fig. 2D); HCC versus cirrhosis: AUC (95%CI) 0.718 (0.622–0.810), sensitivity 53.3%, specificity 100% (Fig. 2E); and HCC versus chronic HBV infection without HCC: AUC (95%CI) 0.721 (0.643–0.790), sensitivity 54.7%, specificity 93.6% (Fig. 2F, Table S6). The addition of AFP to PTX3 significantly increased the performance of PTX3 for discriminating AFP positive HCC from cirrhosis [AUC (95%CI) 0.931 (0.865–0.971), sensitivity 81.33%, specificity 100%, Fig. 2E] or chronic HBV infection without HCC [AUC (95%CI) 0.942 (0.892–0.973), sensitivity 81.3%, specificity 96.2%, Fig. 2F, Table S6].

**Figure 3:**
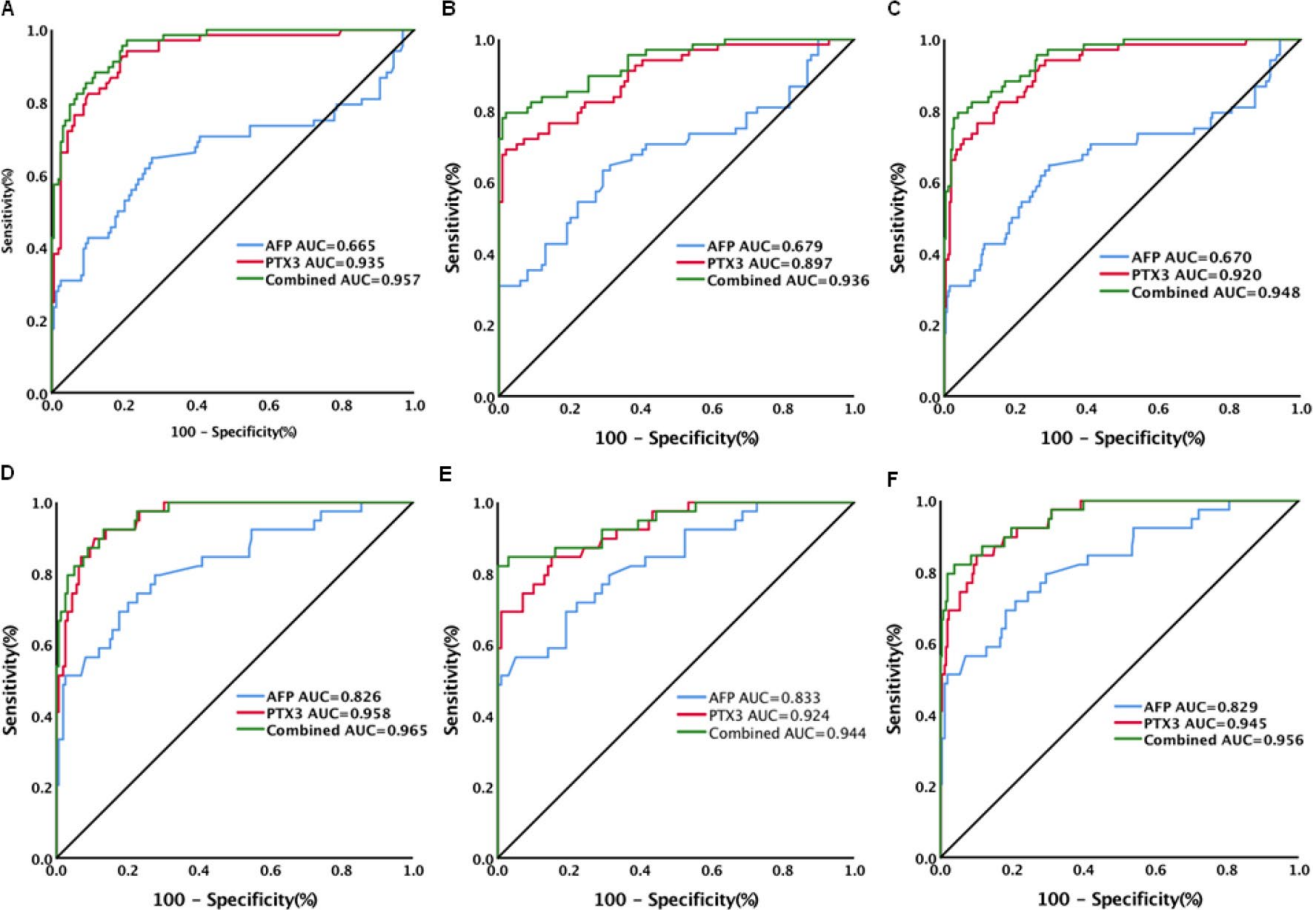
Diagnostic performance of serum PTX3, AFP and combination of PTX3 with AFP for early and late hepatocellular carcinoma (HCC) in patients with chronic HBV infection. (**A**) Diagnostic performance of serum PTX3, AFP and combination of PTX3 with AFP for early HCC from chronic hepatitis; (**B**) Diagnostic performance of serum PTX3, AFP and combination of PTX3 with AFP for early HCC from cirrhosis; (**C**) Diagnostic performance of serum PTX3, AFP and combination of PTX3 with AFP for early HCC from patients without HCC (chronic hepatitis and cirrhosis); (**D**) Diagnostic performance of serum PTX3, AFP and combination of PTX3 with AFP for late HCC from chronic hepatitis; (**E**) Diagnostic performance of serum PTX3, AFP and combination of PTX3 with AFP for late HCC from cirrhosis; (**F**) Diagnostic performance of serum PTX3, AFP and combination of PTX3 with AFP for late HCC from patients without HCC (chronic hepatitis and cirrhosis).

According to HCC stages (Table S7), PTX3 levels also remained highly discriminative of early-stage HCC from chronic hepatitis [AUC (95%CI) 0.935 (0.894–0.963), sensitivity 94.1%, specificity 79.3%, Fig. 3A], cirrhosis [AUC (95%CI) 0.897 (0.840–0.938), sensitivity 69.1%, specificity 98.0%, Fig. 3B] and chronic HBV infection without HCC [AUC (95%CI) 0.920 (0.885–0.947), sensitivity 76.5%, specificity 90.70%, Fig. 3C, Table S8]. The corresponding results of AFP were: HCC versus chronic hepatitis: AUC (95%CI) 0.665 (0.599–0.726), sensitivity 64.7%, specificity 72.3% (Fig. 3A); HCC versus cirrhosis: AUC (95%CI) 0.679 (0.603–0.749), sensitivity 63.2%, specificity 70.7% (Fig. 3B); and HCC versus chronic HBV infection without HCC [AUC (95%CI) 0.670 (0.616–0.721), sensitivity 64.7%, specificity 70.5%, Fig. 3C, Table S8). The combination of PTX3 with AFP did not improve the performance of PTX3 for differentiating early-stage HCC from chronic hepatitis (AUC 0.957, Fig. 3A) but improved the performance for discriminating early-stage HCC from cirrhosis [AUC (95%CI) 0.936 (0.888–0.968), sensitivity 79.4%, specificity 98%, Fig. 3C] or chronic HBV infection without HCC [AUC (95%CI) 0.948 (0.918–0.970), sensitivity 79.4%, specificity 96.1%, Fig. 3C, Table S6]. For late-stage HCC, PTX3 levels also remained highly discriminative of HCC from chronic hepatitis [AUC (95%CI) 0.958 (0.920–0.981), sensitivity 89.7%, specificity 89.3%, Fig. 3D], cirrhosis [AUC (95%CI) 0.924 (0.867–0.962), sensitivity 84.6%, specificity 84.9%, Fig. 3E] and chronic HBV infection without HCC [AUC (95%CI) 0.945 (0.913–0.968), sensitivity 84.6%, specificity 89.9%, Fig. 3F, Table S8]. The corresponding results of AFP were: HCC versus chronic hepatitis: AUC (95%CI) 0.826 (0.766–0.876), sensitivity 79.5%, specificity 72.3% (Fig. 3D); HCC versus cirrhosis: AUC (95%CI) 0.833 (0.760–0.891), sensitivity 56.4%, specificity 95.0% (Fig. 3E); and HCC versus chronic HBV infection without HCC: AUC (95%CI) 0.829 (0.781–0.870), sensitivity 69.2%, specificity 81.8% (Fig. 3F, Table S8). The combination of PTX3 with AFP, although slightly increased AUC values, did not significantly increase the performance of PTX3 for discriminating late-stage HCC from chronic hepatitis (AUC 0.965), cirrhosis (AUC 0.944) or chronic HBV infection without HCC (AUC 0.956, Fig. 3, Table S8).

## Discussion

This study demonstrated significantly elevated serum PTX3 levels in patients with chronic HBV infection compared to healthy controls and in patients with HBV-related HCC compared to patients with chronic hepatitis or cirrhosis. High PTX3 level was an independent risk factor of HCC. The PTX3 levels in HCC patients appeared to be related to HCC differentiation, with low differentiation HCC having higher PTX3 levels in comparison to high differentiation HCC. Importantly, serum PTX3 levels could highly discriminate HCC from chronic hepatitis, cirrhosis and chronic HBV infection without HCC. The discrimination of HCC by PTX3 levels was not influenced by the underlying disease (chronic hepatitis or cirrhosis) or the usage of antiviral treatment with nucleos(t)ide analogues. In particular, PTX3 levels were highly discriminative of AFP-negative (**≤ **20 ng/mL) and early-stage HCC from chronic hepatitis, cirrhosis and chronic HBV infection without HCC. These findings suggest that PTX3 may act as a major contributing factor to the progress of HBV disease and the development of HCC. The determination of PTX3 levels may have a significant diagnostic value for HBV-related HCC including AFP-negative and early-stage HCC in chronic HBV infection.

PTX3 has been revealed to be an extrinsic oncosuppressor in preclinical models and certain tumors through regulating complement-driven macrophage-mediated tumor progression and tuning cancer-related inflammation^22,25,40,41^. However, it remains unknown whether PTX3 plays a protective or promotive role in cancer in that it may impose an important influence on various aspects of cancer such as tumor initiation, angiogenesis, metastasis and immune-regulation^42^. Elevated PTX3 expression has been shown to be associated with poor prognosis in certain cancers, such as breast cancer^25^, gastric cancer^26^, lung cancer^27^, pancreatic cancer^28^ and prostate cancer^29^. Elevated PTX3 plasma level was a risk factor for HCC occurrence in chronic HCV infection^35^ and higher PTX3 expression in tumor tissues was also related to poor prognosis in HCC patients^36^. Consistently, this study showed that elevated circulating PTX3 levels were related to HCC development in chronic HBV infection.

The mechanisms by which PTX3 could be related to the occurrence and development of HCC are largely unknown. There are several potential explanations. First, PTX3 plays a complicated regulatory role in cancer-related inflammation^22,24^. In liver diseases, PTX3 levels were markedly higher in NASH than in simple steatosis non-NASH patients^30,31^. PTX3 levels were also related to the severity of liver fibrosis in NASH^30^ and HCV infection^34^. It is suggested that PTX3 is involved in pathogenesis of hepatic inflammation and fibrosis which are common milieu at the origin of HCC^43^. Second, the involvement of PTX3 in immune response may also play a role in its association with HCC. Expression of PTX3 is inducible by tumor necrosis factor (TNF)-α and interleukin (IL)-1^44^. Differentialy expressed TNF-α from HBV-specific CD8 + T cells was associated with the outcomes of chronic HBV infection including asymptomatic status, active chronic hepatitis and HBV-related HCC^45^. IL-1β levels were significantly and progressively increased with the disease advancement to HCC in chronic HBV infection^46^. Additionally, IL-10 could stimulate B cells in adaptive immunity through enhancing PTX3 production^20^. Elevated IL-10 was suggested to mediate disease progress, from inactive state to cirrhosis and HCC in HBV infection^47^. HCC patients also exhibited significantly higher frequencies of IL-10-expressing B cells that could suppress cytotoxic CD4+ T cell function related to poor survival and high recurrence rate of HCC^48^. Third, although PTX3 is believed not to be produced by hepatocytes^49^, it was shown to be able to enhance HCC cell proliferation and to induce epithelial-mesenchymal transition (EMT)^36^, a biologic process closely related to tumor cell invasion and metastasis.

An important factor for improving long-term prognosis of HCC patients is to detect and treat the tumor at its early stage^7,9^. AFP is the most commonly used biomarker but it is not suitable to detect early stage^11^ and AFP-negative HCC^12^. Therefore, identification of novel biomarkers for HCC remains an urgent need. In the present study, PTX3 was highly discriminative of HCC in chronic HBV infection. In particular, PTX3 was highly and accurately discriminative of AFP-negative and early-stage HCC and the diagnostic performance of PTX3 was superior to AFP. These findings suggest the potentail of PTX3 to be used as a more sensitive biomarker for HCC including early HCC and as a supplemental biomarker for AFP-negative or low AFP-displaying HCC in chronic HBV infection.

For diagnostic accuracy, PTX3 exhibited higher AUC, sensitivity, and specificity than did AFP in HCC patients in relation to chronic hepatitis, cirrhosis and chronic HBV infection without HCC. Detecting of both PTX3 and AFP improved the diagnostic accuracy for HCC in comparison with either detection alone. Although the addition of AFP to PTX3 did not significantly increase the diagnostic ability of PTX3 alone for AFP-negative HCC, the combination of PTX3 with AFP improved diagnostic accuracy when only early-stage HCC was evaluated.

Addition of AFP to ultrasound was shown to significantly increase sensitivity of early HCC detection^50^. PTX3 exhibited good diagnostic performance for the detection of early HCC from HBV chronically infected populations. Whether addition of PTX3 to ultrasound can improve the sensitivity of HCC detection deserves further investigation. Moreover, this study showed that the addition of PTX3 to AFP may allow further identification of HCC in the HBV-related liver diseases. Whether addition of both PTX3 and AFP to ultrasound can further improve the ability and accuracy of HCC detection also deserves investigation. Moreover, serum des-gamma-carboxy prothrombin (DCP) was revealed to be an ideal marker for the diagnosis of HBV-related HCC^51^. We did not compare PTX3 and DCP in the study because measurement of DCP was not carried out in this study. The comparison of PTX3 and DCP is an interesting issue to be addressed in future studies.

We recognized some limitations of the study. First, this is a retrospective study in a relatively small number of patient population with cross-sectional diseases of chronic HBV infection and did not examine the changes of PTX3 levels before and after HCC development. Second, the lack of a validation population may affect the strength of the study. Third, this study only included patients with chronic HBV infection and patients with other etiologies were not included for investigation and comparison. This limits the generalization of the findings to disease conditions of other etiologies such as chronic HCV infection and NASH. Nevertheless, this study demonstrated consistent findings of the diagnostic accuracy of PTX3 for HCC in various disease conditions of chronic HBV infection, and both multivariable analysis and the analysis of data after PSM exhibited the independent role and diagnostic potential of PTX3 in HBV-related HCC, providing evidence for further studies of enhancing and extending the diagnostic potential of PTX3 in HCC detection and optimization of HCC surveillance.

## Conclusion

This study demonstrated the differences of circulating PTX3 levels in various diseases in chronic HBV infection and identified serum PTX3 as an independent factor associated with HCC. Particularly, PTX3 can be used as a new indicator for HCC diagnosis. The performance of PTX3 for differentiating HCC from chronic HBV infection is superior to AFP. Furthermore, PTX3 is highly and accurately discriminative of AFP-negative and early-stage HCC. The combination of PTX3 with AFP may allow further identification of HCC in the HBV-related liver disease population. Therefore, PTX3 may be used as a simple and potentially readily available test that can accurately discriminate HCC in chronic HBV infection. Prospective studies with large patient populations of various etiologies are warranted to validate these results and to directly compare PTX3 with other noninvasive markers before establishing its diagnostic value.

### Ethics approval

The study was conducted under approval of Ethics Committee of the First Affiliated Hospital of Xi’an Jiaotong University. All experiments were conducted in accordance with the Declaration of Helsinki. All the participants provided written informed consent to participate in the study.

## Supplementary information

Supplementary Information.

## Data Availability

The data generated and analysed during this study are available from the corresponding author on reasonable request.
