# Supplementary material for: Serum pentraxin 3 as a biomarker of hepatocellular carcinoma in chronic hepatitis B virus infection
Source: Sci Rep. 2020 Nov 20;10:20276. doi: 10.1038/s41598-020-77332-3 (PMC7680106; doi:10.1038/s41598-020-77332-3)
Supplement: Supplementary file 1 — Supplementary Information. [file 41598_2020_77332_MOESM1_ESM.pdf]

Serum pentraxin 3 as a biomarker of hepatocellular carcinoma in chronic hepatitis B virus infection

Huan Deng, Xiude Fan, Xiaoyun Wang, Lu Zeng, Kun Zhang, Xiaoge Zhang, Na Li, Qunying Han, Yi Lv, Zhengwen Liu

Supplementary materials

Table S1. Demographic and laboratory parameters in the patients with different clinical diseases of chronic HBV infection before and after propensity score matching.

Chronic hepatitis *vs.* HCC

	Befor propensity score matching			After propensity score matching		
	Chronic hepatitis (n=159)	HCC (n=107)	*P*	Chronic hepatitis (n=44)	HCC (n=44)	*P*
Gender			<0.001			0.461
Male,n(%)	96(60.4%)	92(86%)		31(70.5)	35(79.5)	
Female,n(%)	63(39.6%)	15(14%)		13(29.5)	9(20.5)	
Age(Years)			<0.001			0.739
≤55,n(%)	149(93.7)	70(65.4)		38(86.4)	40(90.9)	
>55,n(%)	10(6.3)	37(34.6)		6(13.6)	4(9.1)	
HBV DNA (IU/mL, log)			0.028			0.588
≤10^4,n(%)	34(21.4)	35(32.7)		16(36.4)	16(36.4)	
>10^4,n(%)	125(78.6)	72(67.3)		28(63.6)	28(63.6)	
ALT (IU/L)			0.448			0.314
≤40,n(%)	56(35.2)	36(33.6)		10(22.7)	13(29.5)	
>40,n(%)	103(64.8)	71(66.4)		34(77.3)	31(70.5)	
AST (IU/L)			0.071			0.052
≤40,n(%)	58(36.5)	29(27.1)		5(11.4)	12(27.3)	
>40,n(%)	101(63.5)	78(72.9)		39(88.6)	32(72.7)	
TBIL (μmol/L)			0.529			0.590
≤40,n(%)	112(70.4)	75(70.1)		30(68.2)	30(68.2)	
>40,n(%)	47(29.6)	32(29.9)		14(31.8)	14(31.8)	
Albumin (g/L)			<0.001			0.500
≤32,n(%)	23(14.5)	48(44.9)		13(29.5)	12(27.3)	
>32,n(%)	136(85.5)	59(55.1)		31(70.5)	32(72.7)	
AFP (ng/mL)			<0.001			0.204
≤200,n(%)	144(90.6)	58(54.2)		38(86.4)	34(77.3)	
>200,n(%)	15(9.4)	49(45.8)		6(13.6)	10(22.7)	
PTX3 (ng/mL)	3.7 (1.6-6.4)	15.6 (9.6-24.3)	<0.001	4.7(1.85-6.91)	19.4(10.9-25.96)	<0.001

Cirrhosis *vs.* HCC

	Before propensity score matching			After propensity score matching		
	Cirrhosis (n=99)	HCC (n=107)	*P*	Cirrhosis (n=61)	HCC (n=61)	*P*
Gender			<0.001			0.500
Male,n(%)	61(61.6)	92(86.0)		48(78.7)	49(80.3)	

Female,n(%)	38(38.4)	15(14.0)		13(21.3)	12(19.7)	
Age(Years)			0.024			0421
≤55,n(%)	78(78.8)	70(65.4)		42(68.9)	44(72.1)	
>55,n(%)	21(21.2)	37(34.6)		19(31.1)	17(27.9)	
HBV DNA (IU/mL, log)			0.195			0.500
≤10^4,n(%)	26(26.3)	35(32.7)		18(29.5)	17(27.9)	
>10^4,n(%)	73(73.7)	72(67.3)		43(70.5)	44(72.1)	
ALT (IU/L)			0.157			0.289
≤40,n(%)	41(41.4)	36(33.6)		26(42.6)	22(36.1)	
>40,n(%)	58(58.6)	71(66.4)		35(57.4)	39(63.9)	
AST (IU/L)			0.252			0.282
≤40,n(%)	32(32.3)	29(27.1)		22(36.1)	18(29.5)	
>40,n(%)	67(67.7)	78(72.9)		39(63.9)	43(70.5)	
TBIL (μmol/L)			0.411			0420
≤40,n(%)	67(67.7)	75(70.1)		43(70.5)	45(73.8)	
>40,n(%)	32(32.3)	32(29.9)		18(29.5)	16(26.2)	
Albumin (g/L)			<0.001			0.234
≤32,n(%)	60(60.6)	48(44.9)		34(55.7)	29(47.5)	
>32,n(%)	39(39.4)	59(55.1)		27(44.3)	32(52.5)	
AFP (ng/mL)			<0.001			0.587
≤200,n(%)	86(86.9)	58(54.2)		48(78.7)	48(78.7)	
>200,n(%)	13(13.1)	49(45.8)		13(21.3)	13(21.3)	
PTX3 (ng/mL)	6.2 (3-9)	15.6 (9.6-24.3)	<0.001	5.95(2.94-9.0)	17.7(9.5-22.9)	<0.001

Non-HCC *vs.* HCC

	Befor propensity score matching			After propensity score matching		
	Non-HCC (n=258)	HCC (n=107)	*P*	Non-HCC (n=80)	HCC (n=80)	*P*
Gender			<0.001			0.083
Male,n(%)	157(60.9)	92(86.0)		60(75.0)	68(85.0)	
Female,n(%)	101(39.1)	15(14.0)		20(25.0)	12(15.0)	
Age(Years)			<0.001			0.500
≤55,n(%)	227(88.0)	70(65.4)		60(75.0)	61(76.3)	
>55,n(%)	31(12.0)	37(34.6)		20(25.0)	19(23.7)	
HBV DNA (IU/mL, log)			0.042			0.033
≤10^4,n(%)	60(23.3)	35(32.7)		33(41.3)	21(26.3)	
>10^4,n(%)	198(76.7)	72(67.3)		47(58.8)	59(73.7)	
ALT (IU/L)			0.277			0.500
≤40,n(%)	97(37.6)	36(33.6)		30(37.5)	21(26.3)	
>40,n(%)	161(62.4)	71(66.4)		50(62.5)	59(73.7)	
AST (IU/L)			0.092			0.500
≤40,n(%)	90(34.9)	29(27.1)		25(31.3)	24(30.0)	
>40,n(%)	168(65.1)	78(72.9)		55(68.7)	56(70.0)	
TBIL (μmol/L)			0.499			0.069
≤40,n(%)	179(69.4)	75(70.1)		46(57.5)	56(70.0)	

>40,n(%)	79(30.6)	32(29.9)		34(42.5)	24(30.0)	
Albumin (g/L)			0.015			0.436
≤32,n(%)	83(32.2)	48(44.9)		31(38.7)	33(41.3)	
>32,n(%)	175(67.8)	59(55.1)		49(61.3)	47(58.7)	
AFP (ng/mL)			<0.001			0.500
≤200,n(%)	230(89.1)	58(54.2)		55(68.7)	54(67.5)	
>200,n(%)	28(10.9)	49(45.8)		25(31.3)	26(32.5)	
PTX3 (ng/mL)	5.07(2.03-7.86)	15.6 (9.6-24.3)	<0.001	4.99(2.38-8.91)	16.5(10.3-25.7)	<0.001

HBV, hepatitis B virus; HCC, hepatocellular carcinoma; ALT, alanine aminotransferase; AST, aspartate aminotransferase; TBIL, total bilirubin; PTX3, pentraxin 3; AFP, α-fetoprotein.

Table S2. Diagnostic performance of serum PTX3, AFP and the combination of PTX3 with AFP levels for HCC in chronic hepatitis, cirrhosis and chronic HBV infection without HCC (CH+LC) after propensity score matching.

	AUC (95% CI)	Cutoff (ng/mL)	Sensitivity (%)	Specificity (%)	Youden index	PPV (%)	NPV (%)	Positive LR	Negative LR
HCC *vs*. CH									
PTX3	0.954 (0.887-0.987)	8.958	90.91	88.64	0.796	88.9	90.7	8.0	0.1
AFP	0.514 (0.405-0.622)	5.00	77.27	2.27	0.205	44.2	9.1	0.79	10
PTX3+AFP	0.959 (0.893-0.990)	-	86.36	95.45	0.818	95	87.5	19	0.14
HCC *vs*. LC									
PTX3	0.914 (0.849-0.957)	10.143	72.13	95.08	0.6721	93.6	77.3	14.67	0.29
AFP	0.602 (0.510-0.690)	16.58	67.21	55.74	0.229	60.3	63	1.52	0.59
PTX3+AFP	0.926 (0.865-0.966)	-	75.41	98.36	0.784	97.9	80	46	0.25
HCC *vs*. CH+LC									
PTX3	0.936 (0.887-0.969)	9.013	87.5	83.75	0.713	84.3	87	5.38	0.15
AFP	0.575 (0.495-0.653)	25.90	27.5	96.25	0.238	88	57	7.33	0.75
PTX3+AFP	0.944 (0.896-0.974)	-	77.5	98.75	0.763	98.4	81.4	62	0.23

PTX3, pentraxin 3; AFP, alpha-fetoprotein; HBV, hepatitis B virus; HCC, hepatocellular carcinoma; CH, chronic hepatitis; LC, liver cirrhosis; AUC, area under receiver operating characteristic (ROC) curve; PPV, positive predictive value; NPV, negative predictive value; LR, likelihood ratio.

Table S3. Characteristics of patients with chronic HBV infection under and not under antiviral treatment.

	Antiviral (n=87)	Non-antiviral (n=278)	*P*
Gender (M/F)	62/25	187/91	0.485
Age (years)	44.5±11.19(18-66)	40.3±14.26(18-78)	0.045
HBV DNA (IU/mL, log)	5.32±1.82	5.51±1.71	0.410
ALT (IU/L)	51(14-1040)	59(7-3629)	0.435
AST (IU/L)	61(19-1022)	64(13-4082)	0.538
TBIL (μmol/L)	26.3(2.7-470)	21.4(1.48-727)	0.344
Albumin (g/L)	33.8(18.8-46.8)	36.4(18.8-51.3)	0.056
AFP (ng/mL)	22.2(1.63-97982)	13.35(1.15-325900)	0.062
PTX3 (ng/mL)	8.57(0.384-56.6)	6.31(0.089-42.7)	0.034

HBV, hepatitis B virus; AFP, α-fetoprotein; HCC, ALT, alanine aminotransferase; AST, aspartate aminotransferase; TBIL, total bilirubin; PTX3, pentraxin 3.

Table S4. Diagnostic performance of PTX3, AFP and the combination of PTX3 with AFP for HCC in chronic HBV infection without HCC (CH+LC) under and not under antiviral treatment.

	AUC (95% CI)	Cutoff (ng/mL)	Sensitivity (%)	Specificity (%)	Youden index	PPV (%)	NPV (%)	Positive LR	Negative LR
**PTX3**									
Antiviral									
HCC vs. CH+LC	0.923 (0.865-0.982)	9.708	80	89.47	69.47	80	89.5	7.6	0.22
Non-antiviral									
HCC vs. CH+LC	0.929 (0.896-0.963)	9.231	79.22	91.54	70.76	78.2	92	9.37	0.23
**AFP**									
Antiviral									
HCC vs. CH+LC	0.731 (0.609-0.852)	965.8	43.33	100.00	43.33	100	77	-	0.57
Non-antiviral									
HCC vs. CH+LC	0.729 (0.653-0.804)	20.3	68.83	74.13	42.94	50.5	86.1	2.66	0.43
**PTX3+AFP**									
Antiviral									
HCC vs. CH+LC	0.947 (0.900-0.994)	-	76.67	98.25	74.91	98.5	88.9	43.7	0.24
Non-antiviral									
HCC vs. CH+LC	0.952 (0.927-0.977)	-	80.52	96.02	76.54	88.6	92.8	20.23	0.2

PTX3, pentraxin 3; AFP, α-fetoprotein; HCC, hepatocellular carcinoma; CH, chronic hepatitis; LC, liver cirrhosis; AUC, area under receiver operating characteristic (ROC) curve; PPV, positive predictive value; NPV, negative predictive value; LR, likelihood ratio.

Table S5. Characteristics of patients with AFP negative (≤20ng/mL) and positive (>20ng/mL) HCC.

Variables	AFP negative (n=32)	AFP positive (n=75)	*P*
Gender (Male,%)	91 (29/32)	84 (63/75)	0.549
Age (year)	49.69±2.14 (23-77)	49.33±1.21 (18-68)	0.878
HBV DNA (IU/mL, log)	4.95±0.28	4.88±0.17	0.883
ALT (IU/L)	44 (29.3-77.5)	56 (35-108)	0.069
AST (IU/L)	46 (29-81.6)	90 (52-186)	0.001
TBIL (μmol/L)	15.4 (12.8-29.8)	32 (19.2-73.5)	0.000
Albumin (g/L)	37.9 (30.9-40.6)	31.6 (28.9-37.4)	0.004
PTX3 (ng/mL)	16 (11-24.8)	14.5 (9.4-29.3)	0.346
BCLC stages (0/A/B/C/D)	5/19/5/1/2	12/32/12/12/7	0.327

HBV, hepatitis B virus; AFP, α-fetoprotein; HCC, ALT, alanine aminotransferase; AST, aspartate aminotransferase; TBIL, total bilirubin; PTX3, pentraxin 3.

Table S6. Diagnostic performance of PTX3, AFP and the combination of PTX3 with AFP for AFP negative (≤20ng/mL) and positive (>20ng/mL) HCC in chronic hepatitis, cirrhosis and chronic HBV infection without HCC (CH+LC).

	AUC (95% CI)	Cutoff (ng/mL)	Sensitivity (%)	Specificity (%)	Youden index	PPV (%)	NPV (%)	Positive LR	Negative LR
**AFP negative HCC**									
HCC vs. CH									
PTX3	0.956 (0.909-0.983)	8.949	90.6	91.2	81.8	74.7	97.2	10.24	0.10
AFP	0.625 (0.540-0.704)	3.43	40.6	86.7	27.4	46.4	83.8	3.06	0.68
PTX3+AFP	0.958 (0.912-0.984)	-	93.8	87.6	81.4	68.2	98	7.57	0.07
HCC vs. LC									
PTX3	0.933 (0.865-0.973)	10.143	81.3	95.6	76.8	89.7	91.5	18.42	0.2
AFP	0.531 (0.429-0.632)	7.64	65.6	51.5	17.1	38.9	76.1	1.35	0.67
PTX3+AFP	0.932 (0.864-0.973)	-	81.3	94.1	75.4	86.7	91.4	13.81	0.2
HCC vs. CH+LC									
PTX3	0.947 (0.908-0.973)	8.985	90.6	86.1	76.7	53.7	98.1	6.53	0.11
AFP	0.587 (0.518-0.654)	7.64	65.6	57.2	22.9	21.4	90.4	1.53	0.6
PTX3+AFP	0.948 (0.909-0.974)	-	84.4	92.2	76.6	65.9	97.1	10.58	0.17
**AFP positive HCC**									
HCC vs. CH									
PTX3	0.927 (0.865-0.966)	9.304	76	93.5	69.5	95	70.5	11.65	0.26
AFP	0.718 (0.629-0.796)	20.36	54.7	91.3	46.0	91.1	55.3	6.29	0.5
PTX3+AFP	0.942 (0.892-0.973)	-	81.3	96.2	77.5	95.3	84.3	21.15	0.19
HCC vs. LC									
PTX3	0.887 (0.810-0.940)	11.719	65.3	100	65.3	100	54.5	-	0.35
AFP	0.718 (0.622-0.810)	20.0	53.3	100	53.3	100	47	-	0.47
PTX3+AFP	0.931 (0.865-0.971)	-	81.33	100	81.33	100	68.9	-	0.19

HCC vs. CH+LC									
PTX3	0.912 (0.855-0.952)	9.168	77.3	89.7	67.1	89.7	80.5	7.54	0.25
AFP	0.721 (0.643-0.790)	20.36	54.7	93.6	48.3	89.1	68.2	8.53	0.48
PTX3+AFP	0.942 (0.892-0.973)	-	81.3	96.2	77.5	95.3	84.3	21.15	0.19

PTX3, pentraxin 3; AFP, alpha-fetoprotein; HBV, hepatitis B virus; HCC, hepatocellular carcinoma; CH, chronic hepatitis; LC, liver cirrhosis; AUC, area under receiver operating characteristic (ROC) curve; PPV, positive predictive value; NPV, negative predictive value; LR, likelihood ratio.

Table S7. Characteristics of patients with early stage and late stage HCC.

Variables	Early (n=68)	Late (n=39)	*P*
Gender (Male,%)	90 (61/68)	79 (31/39)	0.143
Age (year)	49±1.40 (18-77)	50±1.56 (36-68)	0.584
HBV DNA (IU/mL, log)	4.87±0.19	4.97±0.23	0.454
ALT (IU/L)	45 (29-100)	63(41-103)	0.080
AST (IU/L)	59 (29.5-128.8)	105 (56-201)	0.003
TBIL (μmol/L)	22.1 (13.5-35.5)	37 (19-112)	0.002
Albumin (g/L)	35 (29.8-40.2)	31 (27.4-36.8)	0.009
AFP (ng/mL)	59 (6.8-2357)	689 (22.6-5814)	0.033
PTX3 (ng/mL)	15 (9.4-22.3)	19 (9.7-28.7)	0.158

HBV, hepatitis B virus; AFP, α-fetoprotein; HCC, ALT, alanine aminotransferase; AST, aspartate aminotransferase; TBIL, total bilirubin; PTX3, pentraxin 3.

Table S8. Diagnostic performance of PTX3, AFP and the combination of PTX3 with AFP for early and late stage HCC in chronic hepatitis, cirrhosis and chronic HBV infection without HCC (CH+LC).

	AUC (95% CI)	Cutoff (ng/mL)	Sensitivity (%)	Specificity (%)	Youden index	PPV (%)	NPV (%)	Positive LR	Negative LR
**Early stage HCC**									
HCC vs. CH									
PTX3	0.935 (0.894-0.963)	7.067	94.1	79.3	73.4	66	96.9	4.53	0.074
AFP	0.665 (0.599-0.726)	20.36	64.7	72.3	37	50	82.7	2.34	0.49
PTX3+AFP	0.957 (0.922-0.979)	-	97.1	79.3	76.3	66.7	98.4	4.68	0.037
HCC vs. LC									
PTX3	0.897 (0.840-0.938)	10.833	69.1	98.0	67.1	95.9	82.2	34.21	0.32
AFP	0.679 (0.603-0.749)	22.22	63.2	70.7	33.9	59.7	73.7	2.16	0.52
PTX3+AFP	0.936 (0.888-0.968)	-	79.4	98	77.4	96.4	87.4	39.31	0.21
HCC vs. CH+LC									
PTX3	0.920 (0.885-0.947)	9.359	76.5	90.70	67.2	68.4	93.6	8.22	0.26
AFP	0.670 (0.616-0.721)	20.36	64.7	70.5	35.3	36.7	88.3	2.20	0.50
PTX3+AFP	0.948 (0.918-0.970)	-	79.4	96.1	75.5	84.4	94.7	20.49	0.21
**Late stage HCC**									
HCC vs. CH									
PTX3	0.958 (0.920-0.981)	8.949	89.7	89.3	79.1	67.3	97.3	8.39	0.11
AFP	0.826 (0.766-0.876)	20.36	79.5	72.3	51.8	41.3	93.5	2.87	0.28
PTX3+AFP	0.965 (0.928-0.986)	-	92.3	86.8	79.1	63.2	97.9	6.99	0.09
HCC vs. LC									
PTX3	0.924 (0.867-0.962)	9.231	84.6	84.9	69.5	68.7	93.9	5.58	0.18
AFP	0.833 (0.760-0.891)	23.10	56.4	95	51.4	81.5	84.7	11.17	0.46
PTX3+AFP	0.944 (0.892-0.976)	-	82.1	100	82.1	100	93.4	-	0.18
HCC vs. CH+LC									
PTX3	0.945 (0.913-0.968)	9.231	84.6	89.9	74.5	55.9	97.5	8.4	0.17
AFP	0.829 (0.781-0.870)	20.36	69.2	81.8	51	36.5	94.6	3.80	0.38
PTX3+AFP	0.956 (0.926-0.976)	-	82.1	96.1	78.2	76.2	97.3	21.17	0.19

PTX3, pentraxin 3; HCC, hepatocellular carcinoma; CH, chronic hepatitis; LC, liver cirrhosis; AUC, area under receiver operating characteristic (ROC) curve; PPV, positive predictive value; NPV, negative predictive value; LR, likelihood ratio.

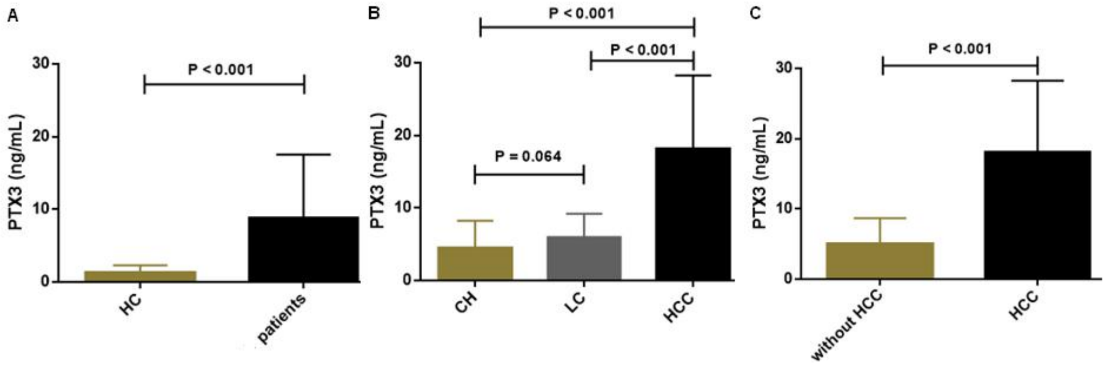

Figure S1. Serum PTX3 levels in patients with chronic hepatitis B virus infection of different disease conditions. **A**. Comparison of PTX3 levels between healthy controls (HC) and patients with chronic HBV infection; **B**. Comparison of PTX3 levels between patients with chronic hepatitis (CH), liver cirrhosis (LC) and hepatocellular carcinoma (HCC); **C**. Comparison of PTX3 levels between patients without and with HCC.

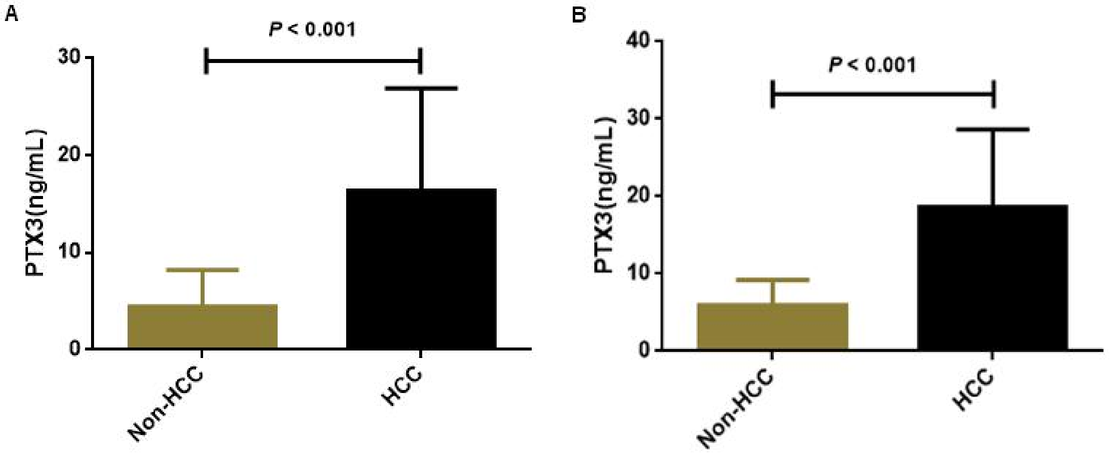

Figure S2. Serum PTX3 levels in patients with hepatocellular carcinoma (HCC) of different underlying liver disease. **A**. Comparison of PTX3 levels between chronic hepatitis patients without and with HCC; **B**. Comparison of PTX3 levels between cirrhosis patients without and with HCC.

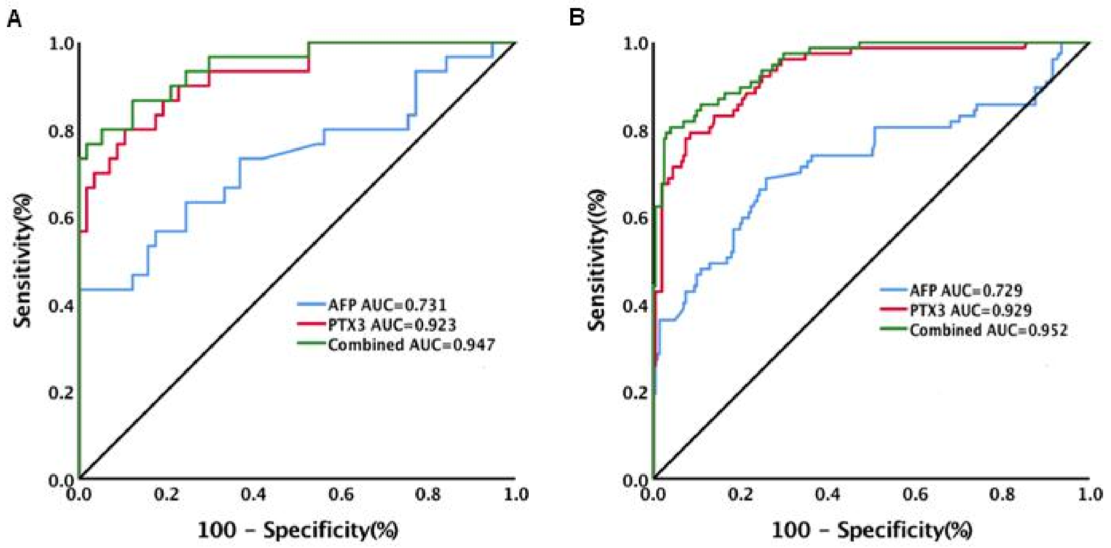

Figure S3. Diagnostic performance of serum PTX3, AFP and combination of PTX3 with AFP for hepatocellular carcinoma (HCC) in patients with chronic HBV infection (chronic hepatitis and cirrhosis) under antiviral treatment and not under antiviral treatment. A. Diagnostic performance of serum PTX3, AFP and combination of PTX3 with AFP for HCC in patients with chronic HBV infection under antiviral treatment. **B**. Diagnostic performance of serum PTX3, AFP and combination of PTX3 with AFP for HCC in patients with chronic HBV infection not under antiviral treatment.
